# A new member of the structured light family: optical spatiotemporal vortices

**DOI:** 10.1038/s41377-023-01281-5

**Published:** 2023-09-16

**Authors:** Fu Feng, Xiaocong Yuan

**Affiliations:** 1 Research Center for Humanoid Sensing, Zhejiang Lab, Hangzhou, 311100 China; 2 Nanophotonics Research Centre, Shenzhen University, Shenzhen, 518060 China

**Keywords:** Transformation optics, Applied optics

## Abstract

The burgeoning growth of structured light has opened up new possibilities for harnessing the spatiotemporal coupling effects in light. Optical spatiotemporal vortices, as a subset of spatiotemporal light, have emerged as a focal point of recent research, owing to their distinctive characteristics and vast range for application. This unique structured light will endow photons with a new degree of freedom, promising to revolutionize researchers’ understanding of photonics. Conducting thorough research on optical spatiotemporal vortices will establish a solid foundation for the development of innovative physical mechanisms and advanced applications in photonics.

## Graphical abstract

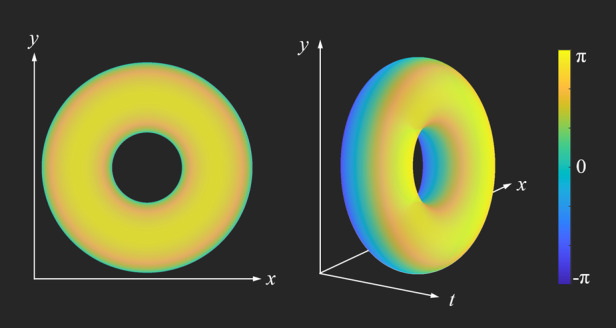

Due to the outstanding contributions of Allen and his team^1^, it has become apparent to researchers that vortex beams possessing a helical phase structure ${e}^{il\theta }$ carry an orbital angular momentum (OAM) of $l\hslash$ per each photon. Considerable research has been conducted to explore the unique properties exhibited by vortex beams. In areas such as optical communication^2^, quantum entanglement^3^, and optical tweezers^4^, vortex beams that possess OAM have demonstrated exceptional promise for applications.

A decade ago, the interest emerged in beams possessing transverse angular momentum. The initial discovery of transverse spin angular momentum (SAM) in beams occurred in strongly focused beams and evanescent waves^5–7^. Analogously, optical vortices carrying transverse OAM have attracted growing interest. Despite the fact that relativity theory predicted the existence of transverse vortex beams^8^, they have yet to be detected in laboratory. This is due to the requirement that an observer must be moving at the speed of light in order to perceive such beams, and as a result, beams with transverse OAM remain unobserved. Thanks to the rapid advancements in the structured light, it has been found that by controlling the spatiotemporal coupling of beams, vortex beams with transverse OAM can be created. Known as spatiotemporal optical vortices (STOV), this unique beam has unveiled a previously unexplored dimension of optics for researchers to investigate.

Recently, eLight published a review of optical spatiotemporal vortices by Professor Qiwen Zhan^9^. This review offers an in-depth look at the principles, properties, and applications of STOVs. By thoroughly analyzing the mathematical expressions and physical interpretations of STOVs, this review provides researchers with fresh insights and a deeper understanding of STOVs, as well as a clear picture of their current state of development and potential future directions.

In the mathematical representation of optical wave fields, the three-dimensional spatial coordinates and one-dimensional time coordinate of light waves are treated as independent for the sake of simplicity. This approach has proven to be both sufficiently accurate and user-friendly for an extended period of time. However, in the case of high intensity and tight focusing^10^, the occurrence of spatiotemporal coupling within the optical wave fields is both inevitable and significant. Indeed, it is the presence of these spatiotemporal coupling effects that imbues the optical wave fields with unique characteristics. Spatiotemporal light sheet, as a typical example of utilizing spatiotemporal coupling effects, can control the propagation characteristics of optical wave packets, group velocity, and even super-beam propagation by changing the distribution on the optical conical surface^11^. The STOV is another quintessential example that takes advantage of the spatiotemporal coupling effect. In contrast to conventional optical vortex that has a spiral phase expressed with two spatial dimensions, the STOVs carries a spatiotemporal spiral phase expressed with one spatial coordinate and one temporal coordinate:

$$E(x,y,z,t)=A(x,y,z,t)\ast \exp (il{\varphi }_{st})\ast \exp [ik(z-vt)],$$

where (*x,y,z*) are three-dimensional spatial coordinates, *t* is one-dimensional time coordinate, *l* is the topological charge, ${\varphi }_{{st}}={\tan }^{-1}\left(\frac{\frac{x}{{x}_{s}}}{\frac{\tau }{{\tau }_{s}}}\right)$ is the azimuthal angle in the spatiotemporal plane, $\tau =z/v-t$ is the local time coordinate, ${x}_{s}$ and ${\tau }_{s}$ are the widths in *x* and $\tau$ directions. The generation of STOVs is remarkably straightforward, necessitating only a two-dimensional pulse shaper^12^. By transforming the incoming pulse onto the spatiotemporal frequency plane via a grating and cylindrical lens, a helical phase can be added using a spatial light modulator (SLM) on this plane, then, being reflected by the original optical path, yields an STOV.

As can be inferred from the aforementioned expression, the spatial coordinates and time coordinates within STOVs are intertwined, giving rise to a spatiotemporal helical phase. The spatiotemporal helical phase is the hallmark of STOVs, with a multitude of their unique attributes arising from this specific helical phase. In contrast to conventional vortex beams, STOVs possess a distinct spatiotemporal topological charge, endowing them with an additional dimension relative to traditional optical wave packets. By leveraging this dimension for encoding purposes, STOVs enjoy benefits in the optical communication and even quantum communication. Due to the presence of phase singularities in the helical phase, the light intensity distribution of STOVs on the spatiotemporal plane exhibits a “dark zone”, making it effortless for STOVs to manipulate small particles. By transferring OAM to particles, nanoparticles can orbit around the singularity line and move with the STOVs^13^.

STOV wave packets come in various forms, each with its own distinct spatiotemporal attributes. Time-varying STOVs are potentially one of the most intriguing forms. By generating multiple spatially separated spatial vortices in the spatiotemporal frequency domain, it is possible to produce multiple temporally separated STOVs within a single wave packet^14^. Each of these STOVs can have a distinct topological charge sign and value. Bessel STOVs have garnered considerable interest among researchers due to their unique properties of propagation invariance and self-healing. Bessel wave packets can be readily generated through the use of spatiotemporal spectral conical phase, with Bessel STOVs being produced following modulation^15^. By employing materials with tailored dispersion parameters or utilizing nonlinear processes to counterbalance the effects of dispersion and diffraction, it is anticipated that Bessel STOVs will exhibit spatial and temporal central lobe propagation invariance over extended distances.

Vortices are a common occurrence in nature, and STOVs represent a new type of optical vortex with topological and conservative properties similar to those of spatial vortices. Their unique ability to carry transverse OAM. STOVs and their variants has demonstrated intriguing photonic properties in a variety of optical phenomena and led to applications in areas such as optical manipulation, spatiotemporal differentiation, pulse propagation, and free-space optical communication. Tremendous research possibilities are still there regarding fully comprehending the physical attributes of these spatiotemporal phase singularities, and exploring their potential applications. Topics of interest may include the propagation of STOVs through optical fibers and slab waveguides, the reflection, refraction, and scattering of STOVs in non-uniform, anisotropic, or nonlinear materials, mode-excitation and light manipulation using STOVs in nanostructures, STOV-assisted dichroism for probing molecular chirality, OPCPA of STOVs, and the characterization and application of ultrafast and ultra-intense STOV pulses in light-matter interaction. It is anticipated that an increasing number of physical mechanisms associated with STOVs will be uncovered and a broad spectrum of applications will be identified in the near future.
